# Feasibility of a Mobile Phone App to Support Recovery From Addiction in China: Secondary Analysis of a Pilot Study

**DOI:** 10.2196/mhealth.8388

**Published:** 2018-02-27

**Authors:** Hui Han, Jing Ying Zhang, Yih-Ing Hser, Di Liang, Xu Li, Shan Shan Wang, Jiang Du, Min Zhao

**Affiliations:** ^1^ Collaborative Innovation Center for Brain Science Shanghai Mental Health Center Shanghai Jiao Tong University School of Medicine Xu Hui District, Shanghai China; ^2^ Los Angeles Integrated Substance Abuse Programs The University of California Los Angeles, CA United States; ^3^ Shanghai Key Laboratory of Psychotic Disorders Shanghai China

**Keywords:** mHealth, substance use, heroin dependence, amphetamine-type stimulant (ATS) dependence, mobile app, China

## Abstract

**Background:**

Mobile health technologies have been found to improve the self-management of chronic diseases. However, there is limited research regarding their feasibility in supporting recovery from substance use disorders (SUDs) in China.

**Objective:**

The objective of this study was to examine the feasibility of a mobile phone-based ecological momentary assessment (EMA) app by testing the concordance of drug use assessed by the EMA, urine testing, and a life experience timeline (LET) assessment.

**Methods:**

A total of 75 participants dependent on heroin or amphetamine-type stimulant (ATS) in Shanghai were recruited to participate in a 4-week pilot study. Of the participants, 50 (67% [50/75]) were randomly assigned to the experimental group and 25 (33% [25/75]) were assigned to the control group. The experimental group used mobile health (mHealth) based EMA technology to assess their daily drug use in natural environments and received 2 short health messages each day, whereas the control group only received 2 short health messages each day from the app. Urine tests and LET assessments were conducted each week and a post-intervention survey was administered to both groups. The correlations among the EMA, the LET assessment, and the urine test were investigated.

**Results:**

The mean age of the participants was 41.6 (SD 8.0) years, and 71% (53/75) were male. During the 4 weeks of observation, 690 daily EMA survey data were recorded, with a response rate of 49.29% (690/1400). With respect to drug use, the percent of agreement between the EMA and the LET was 66.7%, 79.2%, 72.4%, and 85.8%, respectively, for each of the 4 weeks, whereas the percent of agreement between the EMA and the urine test was 51.2%, 65.1%, 61.9%, and 71.5%, respectively. The post-intervention survey indicated that 46% (32/70) of the participants preferred face-to-face interviews rather than the mHealth app.

**Conclusions:**

This study demonstrated poor agreement between the EMA data and the LET and found that the acceptance of mHealth among individuals with SUDs in China was not positive. Hence, greater efforts are needed to improve the feasibility of mHealth in China.

## Introduction

Substance use disorder (SUD) is a major public health issue, not only in China but worldwide as well [1,2]. Based on the report from the United Nations Office on Drugs and Crime (UNODC), approximately 290 million people worldwide use illicit drugs, and marijuana heads the list as the most popular drug used throughout the world [3]. China, the largest developing country, faces serious drug problems. Between 2000 and 2016, the number of registered people in China with a drug addiction increased sharply from 0.86 to 2.50 million [4]. Although the number of users of club drugs, such as methamphetamine, ecstasy, and ketamine, has increased since the last decade, heroin remains a major drug problem, with 49.3% of people registered with a drug addiction being addicted to it [5]. To control the problems of heroin abuse and HIV and AIDS infection, harm-reduction programs, including methadone maintenance treatment (MMT) clinics, have been established throughout China beginning in 2004 [6,7]. By the end of 2014, there were more than 300 MMT clinics providing services to 344,254 addicts [8]. Although the effectiveness of MMT has been demonstrated, its implementation still faces certain problems, including the high cost of MMT, a lack of psychotherapy, and the low dosage of methadone, which contributes to high rates of drop-out and relapse [9,10]. Together, these conditions suggest the need to develop new intervention strategies that meet the needs of people with drug addiction in China [11,12].

A large body of studies has demonstrated that drug addiction is a chronic relapsing brain disease that features cycles of relapse and remission [13]. A chronic condition means that those with SUDs require a chronic care model that can provide an integrated care system that includes services and self-management tools designed to prevent relapse [14,15].

Mobile health (mHealth) service is a new technology that has been widely used in the health care service field as well as in substance abuse treatment programs in western countries [16]. Service providers integrate a tailored mHealth self-management app for patients and collect data from the app to help them monitor their patients’ behaviors [17]. For example, a mobile phone can be programmed to send a message when people with a drug addiction enter a high-risk area that can lead to relapse. Other functions include improving disease management, delivering therapeutic interventions, and increasing healthy behavior [18].

Recently, the mobile phone-based ecological momentary assessment (EMA) began to be used in the field of medical treatment. The EMA is a mHealth technology that is capable of collecting individuals’ data in real time. Compared to other retrospective surveys, the EMA is thought to substantially improve the accuracy of reporting since self-reporting sometimes requires participants to recall events over long periods, a situation that may introduce a systematic bias [19,20].

A few studies have compared the accuracy of the EMA with other measures to assess drug use behaviors. Kranzler [21] assessed the amount of drinking SUDs using a timeline follow-back method (TLFB) and a daily interactive voice recording (IVR) and found poor agreement between the two approaches. Similarly, Searles and Lincoln’s results revealed poor agreement between daily drinking recall and IVR measures [22,23]. Conversely, other researchers [24-26] conducted a set of outcome-based analyses using both the TLFB and the IVR drinking outcomes and found no differences between the two measures.

To our knowledge, there are no published studies that address the feasibility and acceptability of this novel technology among individuals with SUDs in China. This lack of evidence hinders the implementation of mHealth in China. To address this knowledge gap, we conducted secondary analyses based on a pilot study of a mobile phone-based intervention designed to support recovery from addiction. The aims of the present study were to test the concordance of drug use collected via the EMA, urine testing, and a retrospective self-report survey instrument, and to investigate the acceptability of mHealth in China. The overall goal was to provide evidence for the future use of mHealth technology in drug relapse prevention.

## Methods

### Sample Recruitment and Study Procedures

Participants were recruited from 3 MMT clinics and the Zi-Qiang social work consortium. The social work consortium is a community-based social work service network with approximately 500 social workers hired from eligible individuals in the community to help clients living within the same community. All participants were recruited through advertisements in the MMT clinics and the social work consortium. Inclusion criteria were (1) aged 18 to 65 years; (2) above primary education; (3) able to use a mobile phone with app capabilities; (4) a newly enrolled patient in an MMT clinic or social work consortium or relapsed (positive urine test) within the past 30 days; (5) met the Diagnostic and Statistical Manual of Mental Disorders-IV (DSM-IV) criteria for heroin dependence or amphetamine-type stimulant (ATS) dependence; and (6) consent to participate in the study. Exclusion criteria were (1) a main substance that was not heroin or ATS; and (2) diagnosis of serious mental illness and inability to complete the evaluation. Protocols for this study were approved by the institutional review board (IRB) of Shanghai Mental Health Center (No 2012-56C2) and the University of California, Los Angeles (12-000809).

From June 2015 to April 2016, 89 clients were screened at baseline. Of the clients, 7 (8% [7/89]) reported that they had no mobile phone, and 5 (6% [5/89]) refused to participate in the study. Thus, 75 participants were recruited and assigned to either the experimental group (n=50) or the control group (n=25), resulting in a 2:1 ratio.

All participants were required to install the app and were given instructions on how to use it. The app for the experimental group included 6 functions: surveys (daily survey and self-initiated survey), messages, settings, profile, craving, and help. The app for the control group included messages, settings, profile, and help. Participants in the experimental group were asked to complete a daily survey on the app and they received 2 health messages every day for 4 weeks. Participants in the control group only received 2 health messages from the app. Demographics, substance abuse histories, and clinical scale data were collected at baseline. Whether participants engaged in drug use was assessed using the EMA app, the urine test, and the LET once a week during the study period.

### Description of the Mobile Health-Based App

The mHealth was developed specifically to help individuals with SUDs achieve and maintain recovery. Thus far, it is the first mobile app designed for people with SUDs in China. The mechanism of the mHealth app is based on cognitive behavior therapy (CBT) and self-determination theory (SDT). CBT emphasizes triggers and coping strategies for relapse prevention, whereas SDT is a theory that motivates people to change and act for themselves. mHealth is combined with CBT and SDT to create 3 main tools: surveys, messages, and cravings, to foster positive behavior and manage behavior. These tools provide timely assessment and intervention and help individuals with SUDs to control their cravings and prevent relapses.

### Surveys

**Figure 1:**
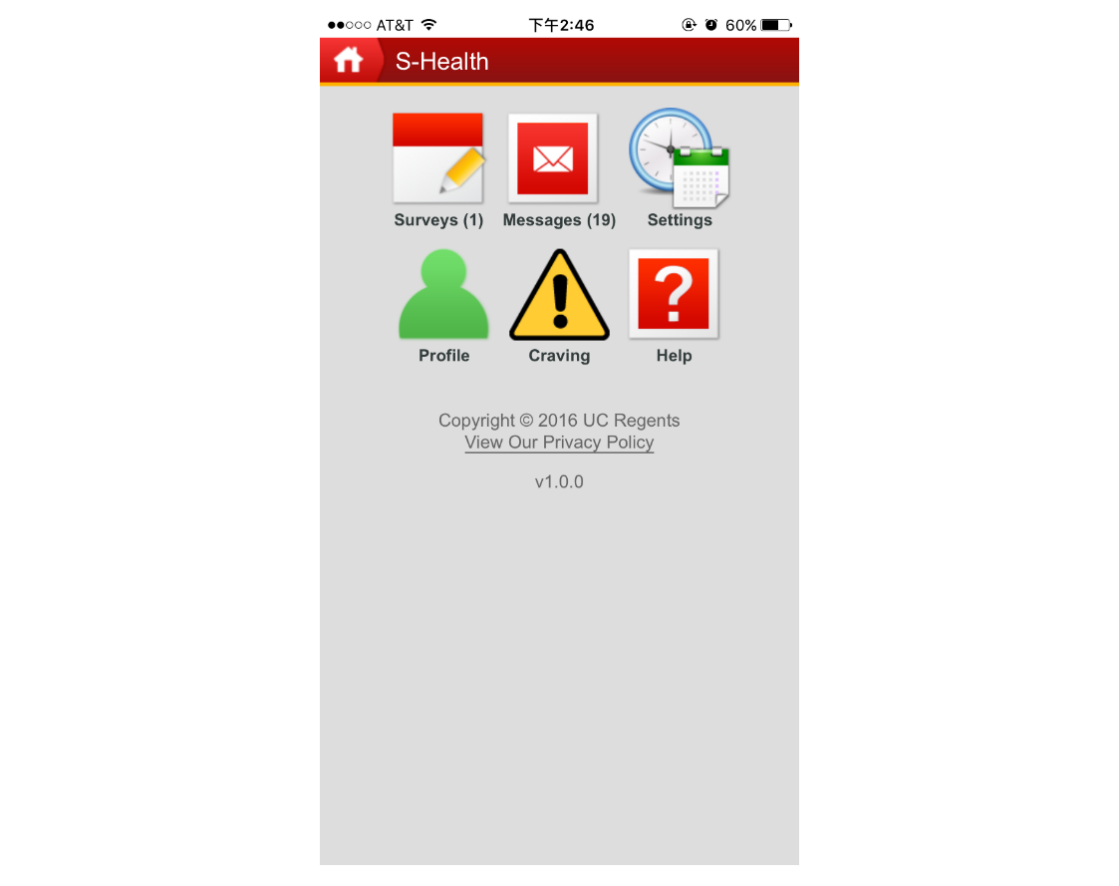
Home screen of the mobile health (mHealth) app.

There are 2 types of surveys built into the app: the daily survey and the self-initiated survey. Participants were alerted to complete the daily survey every 24 hours. This survey required them to report their alcohol, tobacco, and drug use as well as their cravings, triggers, emotions, coping strategies, and daily goal progress for the last 24-hour period. Once participants completed the daily survey, it was no longer available to the participant until the next day at the scheduled time, which was set by the participant. It took 1 to 2 minutes to complete the daily survey. The self-initiated survey was available to participants at any time. The questions on this survey addressed cravings, triggers, and drug use. Once the participant indicated a craving for a drug, he or she could complete the survey and received messages to help control the craving, to a certain extent. A screenshot of the mHealth app home screen is shown in Figure 1.

### Data Collection

#### Ecological Momentary Assessment

The EMA data were collected from the experimental group using the survey function in the app. The daily situation (eg, drug use, craving, coping) was collected by the daily survey, which was conducted every day at a scheduled time. Before the scheduled time, a reminder message was sent to the participants to remind them to complete the daily survey. Craving status was collected by the self-initiated survey, which could be completed any time the participant experienced a craving for a drug. A guide message was then sent to the participant to help control the craving.

#### Life Experience Timeline Assessment

The LET was developed by the University of California, Los Angeles (UCLA) and exhibited good reliability and validity [27,28]. The questionnaire consists of 20 items that record 20 events (eg, substance use, emotion, coping, and craving) over the past week. Participants were requested to complete the LET survey at baseline and each week during the study period. The outcomes collected by the LET indicated whether the individual used any of the identified substances in the last week and, if so, how many days they used drugs in last week.

#### Urine Test

The urine test board was used to test drug use at baseline and each week during the study period. The test identified heroin, ATS, marijuana, cocaine, and ketamine use.

#### Post-Intervention Survey

To measure the acceptability of the electronic mobile (e-mobile) method among individuals with SUDs, the participants were asked to respond “agree” or “disagree” for each of the following items:

It is easy for me to understand these questions.

I feel okay when I answer questions about drug use.

I can recall the days I used drugs during the last week.

It is fine for me to answer the same questions each year.

This mHealth app is easy to use.

Compared to a face-to-face interview, I prefer to use the mHealth app.

An informal group discussion was conducted with 7 participants who voluntarily attended the discussion. The main purpose of the group discussion was to collect supplementary information as to why individuals with SUDs did not want to use the mHealth app.

### Data Analysis

All statistical analyses were conducted using the Statistical Package for Social Science (SPSS, version 22.0). The differences in characteristics between the experimental group and the control group were analyzed using the *t* test for continuous measures and the chi-square test for categorical measures. Statistical significance was set at alpha=.05. Cohen kappa was conducted to measure the correlations among the 3 measures. Concordance correlation coefficients less than 0.20 were regarded as poor, 0.21 to 0.40 as fair, 0.41 to 0.60 as moderate, 0.61 to 0.80 as good, and 0.81 to1.00 as very good. The chi-square test was used to compare the consistency of the EMA and the LET data. A logistic regression was conducted to explore the factors that influenced the correspondence between the EMA and the urine test during the study period. Only non-missing data during the whole week were included in the analysis.

## Results

Baseline characteristics of the participants of the experimental and control groups are shown in Table 1. The mean age of the participants was 41.6 (SD 8.0) years, and 71% (53/75) of the participants were male. Among the experimental group, 76% (38/50) were heroin users and 24% (12/50) were ATS users, whereas among the control group, 80% (20/25) were heroin users and 20% (5/25) were ATS users. There were no significant differences between the groups in terms of the type of drug, age, gender, education, marriage, employment status, initial age of use drug, or length of main substance used. Drug use test results are summarized in Table 2.

**Table 1:** Baseline characteristics of the participants (N=75).

Characteristic		Total	Experimental group (N=50)	Control group (N=25)	t/F/Z	P*
Age, mean (SD)		41.6 (8.0)	41.7 (8.7)	41.3 (6.8)	0.235	.815
Male, n (%)		53 (71)	35 (70)	18 (72)	0.138	.858
**Education, n (%)**					0.538	.463
	≤Middle school	32 (43)	23 (46)	9 (36)		
	≥High school	43 (57)	27 (54)	16 (64)		
**Marital status, n (%)**					0.001	.972
	Married	32 (43)	20 (40)	12 (48)		
	Single	43 (57)	30 (60)	13 (52)		
**Employment, n (%)**					2.600	.107
	Employed	45 (61)	33 (67)	12 (48)		
Initial age of use drug, mean (SD)		26.1 (8.9)	25.9 (8.8)	26.4 (9.2)	-0.211	.834
Length of main drug used (months), mean (SD)		15.5 (6.0)	15.8 (6.1)	14.9 (6.0)	0.615	.541

*Significant at *P*<.05.

**Table 2:** Drug use test results by week.

Test		First week (n)	Second week (n)	Third week (n)	Fourth week (n)
**Urine test**					
	Use	24	21	15	11
	No use	19	22	27	31
	Total	43	43	42	42
**Self-report LET^a^**					
	Use	15	12	10	7
	No use	33	36	38	41
	Total	48	48	48	48
**EMA^b^**					
	Use	12	10	6	5
	No use	28	25	26	25
	Total	40	35	32	30

^a^LET: life experience timeline.

^b^EMA: ecological momentary assessment.

**Figure 2:**
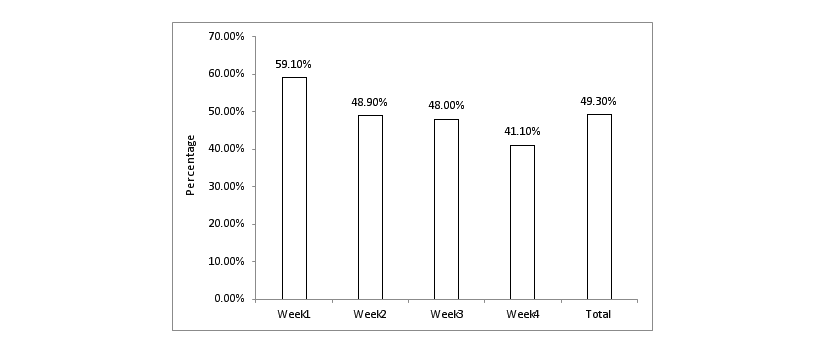
The weekly ecological momentary assessment (EMA) data in the experimental group.

### Ecological Momentary Assessment Data Description

The EMA data were collected from the experiment group. A total of 350 EMA daily surveys were expected each week (50 times 7). The daily survey data received was 59.1% (207/350) the first week; 48.9% (171/350) the second week, 48.0% (168/350) the third week, and 41.1% (144/350) the fourth week. The average response rate was 49.29% (690/1400). These results indicated that the daily survey response rates were generally low (Figure 2).

### Methods of Measuring Drug Use

**Figure 3:**
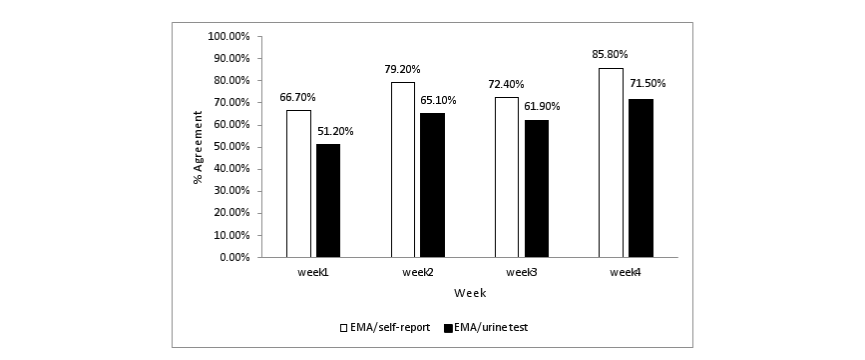
The concordance of test results.

Three methods (ie, EMA, LET, and urine test) were used to assess drug use each week. The correspondence between the EMA and the LET over the 4 weeks was 67% (32/48), 79% (38/48), 72% (35/48), and 86% (41/48), respectively. The correspondence between the EMA and the urine test was 51% (22/43), 65% (28/43), 62% (26/42), 72% (30/42), respectively (Figure 3). The agreement between the EMA and the LET was not good, with Cohen kappas of 0.128, 0.412, 0.111, and 0.241, respectively. With respect to the agreement between the EMA and the urine test, the results were not optimistic according to Cohen kappas, which were 0.076, 0.292, 0.017, and 0.103, respectively. The logistic regression analysis revealed that work and age were correlated with correspondence between the EMA and the urine test during the study period (odds ratio [OR]=0.167, 1.137, 0.195; *P*<.05), as shown in Table 3 and Figure 3.

### Post-Intervention Survey

The post-intervention survey was conducted with all participants to evaluate their attitude toward the mHealth-based app (Table 4). Even though most of the participants (72% [51/71]) admitted that the mHealth app was easy to use, almost half of them (46% [32/70]) stated that compared to the mHealth data collection method, they preferred a face-to-face interview.

**Table 3:** Logistic regression of concordance between the ecological momentary assessment and the urine test.

Week	Factor	β	SE	Chi-square	*P*	OR^a^	95% CI
1	Job	-1.792	0.755	5.625	.018	0.167	0.038-0.733
2	Age	0.129	0.048	7.074	.008	1.137	1.034-1.251
3	Job	-1.636	0.854	3.670	.034	0.195	0.037-1.038

^a^OR: odds ratio.

**Table 4:** Post-intervention survey results.

Item	Agree, n (%)	Disagree, n (%)
1. It is easy for me to understand these questions^a^.	44 (67)	22 (33)
2. I feel comfortable when I answer these questions^b^.	49 (69)	22 (31)
3. I can recall the days I used drugs during the last week^b^.	42 (59)	29 (41)
4. I prefer to answer these questions each year^b^.	51 (72)	20 (28)
5. The mHealth app is easy to use^b^.	51 (72)	20 (28)
6.Compared to a face-to-face interview, I prefer to use the mHealth app^c^.	38 (54)	32 (46)

^a^N=66.

^b^N=71.

^c^N=70.

## Discussion

### Principal Findings

The purpose of this study was to investigate the feasibility and acceptability of mHealth among individuals with SUDs in China. The findings from our study demonstrated that the veracity and acceptability of the EMA was not optimistically received among those with SUDs in China.

In China, people with drug addiction are treated as immoral. Poor adherence to treatment is the primary problem when dealing with substance-related issues [29] because it leads to relapse. Schomerus [30] found that the stigma associated with alcohol dependence was higher than it was for other mental disorders in general population studies and created a barrier for people to routinely enter treatment [31,32]. Thus, mHealth-based data collection methods hold a unique place among people with drug addiction. Nonetheless, our findings indicated that the response rate for EMA data collection was low, with an average response rate of 49.29% (690/1400), and half of the participants preferred face-to-face interviews. The reasons for the low response rate were drawn from the post-intervention survey. Patients who were unwilling or hesitant to use mHealth expressed concerns with privacy. Some mentioned that the mHealth-based app was not safe and they were afraid of being arrested because they thought the police could obtain information about their relapse from the app. They also expressed concerns about the Global Positioning System (GPS) function of the app, claiming that it made them uncomfortable. Moreover, because most of the individuals with SUDs were isolated from society and their relatives, they preferred the face-to-face interviews because they perceived them as a way to communicate with the public. Another factor that affected the response rate is age [33]. Compared to older patients, young patients are more inclined to accept novel technologies, such as mHealth. Conversely, older patients are more conservative and tend to be more concerned about their privacy. Although age was not mentioned as a barrier in this study, it should be considered in future research and in clinical applications.

Another aim of the study was to explore the veracity of the EMA data. The EMA aims to minimize recall bias, maximize ecological validity, and enable the study of behavior in the real world. Our study results indicated that the correlations between the EMA and the LET and the EMA and the urine test were poor. These results are similar to those of previous studies. For example, Pearson [34] compared the accuracy of self-reported electronic cigarette (e-cigarette) puff counts using the EMA to objective puff count data collected by a Bluetooth-enabled e-cigarette device and found poor agreement between the device data and the self-reported data. Solhan [35] examined the discrepancies among trait questionnaires, retrospective reports, and EMA measures of affective instability in psychiatric outpatients and found poor agreement between recalled mood changes and the EMA. Griffith [36] also found poor agreement between the TLFB and the EMA with respect to heavy smokers.

Regarding the impact factors, Patrick [37] found that gender moderated the 3 methods of data collection. Our work indicated that job and age were predictors of the correspondence between the EMA and the urine test. With respect to the influence of employment, people with a job were found to exhibit better correspondence between the EMA and the urine test. This may be because people who have jobs are better able to cooperate with others and are more likely to engage in honest communications and receive assistance from others.

### Limitations

Limitations to our study included a convenience sample that may not be representative of other areas in China or of foreign countries. However, our findings may still be beneficial to those with similar situations. Second, self-reporting and the response to the text message could have been inaccurate due to a number of factors. Third, the low response rate to the EMA daily diaries indicated poor acceptance of mHealth among individuals with SUDs. A future study could combine pre-intervention education and EMA technology to improve the response rate. Finally, because the technology was limited, we were unable to provide accurate feedback or interventions to participants when a craving arose. Despite these limitations, the study presents valuable information regarding the feasibility of electronic health (e-health) among individuals with SUDs.

### Conclusions

Mobile phones have been widely used among the Chinese population, with 1.3 billion mobile phone users in 2016. Thus, mobile phones provide a potential opportunity for the development of mHealth in China. The current study demonstrated poor agreement among the data from the EMA, the LET assessment, and urine testing. Hence, the acceptance of mHealth among individuals with SUDs in China was not enthusiastic. This indicates that more work is needed to optimize the mHealth app and improve its acceptance among individuals with SUDs in China as well as in other countries that are experiencing similar issues.

## Abbreviations

ATS: amphetamine-type stimulant
CBT: cognitive behavior therapy
e-cigarette: electronic cigarette
EMA: ecological momentary assessment
IVR: interactive voice recording
LET: life experience timeline
mHealth: mobile health
MMT: methadone maintenance treatment
SDT: self-determination theory
SUD: substance use disorder
TLFB: timeline follow-back method
